# Improved Assembly of Reference Genome Fusarium oxysporum f. sp. lycopersici Strain Fol4287

**DOI:** 10.1128/MRA.00910-18

**Published:** 2018-09-13

**Authors:** Dilay Hazal Ayhan, Cristina López-Díaz, Antonio Di Pietro, Li-Jun Ma

**Affiliations:** a Department of Biochemistry and Molecular Biology, University of Massachusetts Amherst, Amherst, Massachusetts, USA; b Department of Genetics, University of Córdoba, Córdoba, Spain; Vanderbilt University

## Abstract

Fusarium oxysporum is a pathogenic fungus that infects hundreds of plant species. This paper reports the improved genome assembly of a reference strain, F. oxysporum f. sp. lycopersici Fol4287, a tomato pathogen.

## Abstract (precis)

Fusarium oxysporum is a pathogenic fungus that infects hundreds of plant species. This paper reports the improved genome assembly of a reference strain, F. oxysporum f.

## ANNOUNCEMENT

Fusarium oxysporum is a filamentous fungus that can infect hundreds of plant species, as well as immunocompromised human patients (1). The reference genome of F. oxysporum was first generated using Sanger sequencing with 6× coverage using a tomato-infecting strain, F. oxysporum f. sp. lycopersici Fol4287 (race 2, VCG 0030) (2). The strain was originally isolated from an infected tomato plant in Murcia by Javier Tello from the University of Almería, Spain (2, 3). It is available from the Fungal Genetics Stock Center (FGSC 9935), NCAUR/USDA (NRRL 34936), and CBS-KNAW (CBS 123668) collections. A comparative study of this reference genome with those of closely related species identified lineage-specific (LS) chromosomes that are rich in transposons and genes related to pathogenicity. The transfer of these LS chromosomes between strains of F. oxysporum was experimentally confirmed to convert a nonpathogenic strain into a pathogen (2, 4–6). These studies enabled the structural and functional partitioning of the F. oxysporum genome, which provides a novel means of dissecting fungal pathogenesis.

Unfortunately, this reference genome has a high level of single-nucleotide-level sequencing errors due to the low sequencing coverage. To improve its quality, we regenerated the whole-genome assembly with increased sequence coverage and combining Illumina and PacBio sequence technologies. Genomic DNA was extracted from the mycelium of Fol4287 (the same isolate that was sequenced before). The DNA library for short reads was prepared with an average 400-bp insert size. The DNA library was sequenced at 66× coverage, using the Illumina HiSeq 2500 platform, into 71-bp paired-end reads. The genomic DNA was sequenced using the PacBio RS II system with 10× subread coverage. FastQC (version 0.11.5) was used to check the quality of all reads. The average base quality of Illumina reads is 36.8, with a 71-bp read length. The PacBio reads have average and maximum read lengths of 6.8 kb and 52 kb, respectively.

The initial assembly was generated via SPAdes version 3.9.1 (7), combining raw Illumina and PacBio reads with default parameters. Quiver in SMRT Analysis (version 2.2.0) (8) was used to polish the assembly based on the PacBio reads. Further polishing was performed by mapping the Illumina reads to the assembly using BWA version 0.7.12 (9). FreeBayes v0.9.10-3-g47a713e (10) was used to identify base variants between the reads and the assembly. Highly confident variant sites were used to correct the assembly using a custom script (available at github.com/d-ayhan/tools). We also used structural variant (SV) callers, GRIDSS version 1.4.1 (11) and Sniffles version 1.0.8 (12), to identify the SVs in the initial assembly. All identified SVs were inspected manually to ensure accuracy. High-confidence merges/splits were integrated into the assembly. The improved assembly was then quality checked by remapping. This process was repeated until no future correction could be identified.

The final assembly is 53.9 Mb, with 499 contigs and an *N*_50_ value of 1.3 Mb. The largest contig size is 5.7 Mb. The GC content is 47.7%. The assembly includes a contig of 52,424 bp that captures the complete mitochondrial DNA and a contig of 7,875 bp of the complete ribosomal DNA sequence (2). In a comparison of the new assembly to the reference assembly of F. oxysporum (assembly GCA_000149955) by BLAST, the contigs that belong to each chromosome were identified, ordered, and oriented within each chromosome. The contigs were divided into three categories, including 11 core chromosomes (C), 4 LS chromosomes (S), and some unmapped contigs (U). Except for chromosomes 1 and 2, each core chromosome was assembled into a single contig GenBank assembly number (GCA_003315725).

### Data availability.

This whole-genome shotgun project has been deposited at DDBJ/ENA/GenBank under the accession number QESU00000000. The version described in this paper is version QESU01000000. The PacBio and Illumina reads are available in SRA under accession numbers SRR7015920 and SRR7690004, respectively.
